# Sentimentality and Nostalgia in Elderly People in Bulgaria and Greece – Cross-Validity of the Questionnaire SNEP and Cross-Cultural Comparison

**DOI:** 10.5964/ejop.v13i1.1202

**Published:** 2017-03-03

**Authors:** Stanislava Yordanova Stoyanova, Vaitsa Giannouli, Teodor Krasimirov Gergov

**Affiliations:** a Department of Psychology, South-West University "Neofit Rilski", Blagoevgrad, Bulgaria; b Bulgarian Academy of Sciences, Sofia, Bulgaria; University of South Wales, Newport, United Kingdom

**Keywords:** elderly people, nostalgia, sentimentality

## Abstract

Sentimentality and nostalgia are two similar psychological constructs, which play an important role in the emotional lives of elderly people who are usually focused on the past. There are two objectives of this study - making cross-cultural comparison of sentimentality and nostalgia among Bulgarian and Greek elderly people using a questionnaire, and establishing the psychometric properties of this questionnaire among Greek elderly people. Sentimentality and nostalgia in elderly people in Bulgaria and Greece were studied by means of Sentimentality and Nostalgia in Elderly People questionnaire (SNEP), created by Gergov and Stoyanova (2013). For the Greek version, one factor structure without sub-scales is proposed, while for the Bulgarian version of SNEP the factor structure had four sub-scales, besides the total score. Together with some similarities (medium level of nostalgia and sentimentality being widespread), the elderly people in Bulgaria and Greece differed cross-culturally in their sentimentality and nostalgia related to the past in direction of more increased sentimentality and nostalgia in the Bulgarian sample. Some gender and age differences revealed that the oldest male Bulgarians were the most sentimental. The psychometric properties of this questionnaire were examined for the first time in a Greek sample of elders and a trend was found for stability of sentimentality and nostalgia in elderly people that could be studied further in longitudinal studies.

This paper has two objectives that are related to each other. One objective is making cross-cultural comparison of sentimentality and nostalgia among Bulgarian and Greek elderly people using a questionnaire (Sentimentality and Nostalgia in Elderly People questionnaire – SNEP, by Gergov & Stoyanova, 2013) in cross-cultural research. In order to achieve this objective of the study, another objective of the study should also be met, that is establishing psychometric properties of SNEP among Greek elderly people, i.e. checking reliability, construct validity, and cross-validity of the questionnaire SNEP in the sample of Greek elderly people, and creating norms on SNEP for the Greek sample of elderly people.

## Establishing the Psychometric Properties of SNEP

Measuring reliability is necessary for deciding about the use of only such data that are stable and consistent. An instrument that is not reliable cannot be applied (see for example Anastasi & Urbina, 2006) that is why reliability of SNEP in the new Greek sample of elderly people should be established.

Cross-validity means validation of a questionnaire for a new sample differing from the one for which this questionnaire has been created (Anastasi & Urbina, 2006, p. 513); a method that is first applied for one sample of a population is later applied for another sample from the population (“Cross-validation”, n.d.) – in this case, the population of elderly people. The items in SNEP have been created for Bulgarian elderly people (Gergov & Stoyanova, 2013) and cross-cultural comparison of sentimentality and nostalgia between Bulgarian and Greek elderly people requires establishing cross-validity of SNEP for Greek elderly people – using SNEP in another sample different from the initial one for which it has been created (Bulgarian elderly people), but a part of the total population of elderly people.

Construct validity means establishing correspondence between the results from a method and a theoretical model - by means of checking the factor structure of the questionnaire, making inter-group comparisons, such as age-group comparisons for example, etc. (Anastasi & Urbina, 2006, pp. 148-149). Construct validity of the questionnaire SNEP in a Bulgarian sample was checked by means of comparing the different social categories of the elderly Bulgarians and finding the factor structure of SNEP (Gergov & Stoyanova, 2013). Cross-cultural comparison of sentimentality and nostalgia in two groups of elderly people in two countries, as well as their comparisons between different social groups could be dealt with also as establishing construct validity of SNEP. Realizing together these both objectives is attempted to avoid redundant publication, because construct validation and cross-validation of the questionnaire in this case mean cross-cultural comparison to be performed.

## Cross-Cultural Comparison of Sentimentality and Nostalgia

Cross-cultural comparison of sentimentality and nostalgia of elderly people has been already made between Bulgarian Roma elderly people and Hungarian elders living in Romania (Stoyanova, Doseva, Gergov, & Virginás-Tar, 2015) that indicates that the questionnaire SNEP could be applied successfully for cross-cultural comparisons and is cross-validated for minority elderly samples.

Research of sentimentality and nostalgia is important because mental commitment to the past happens to every human being at different ages. However, this problem has been rarely studied by the researchers, especially in cross-cultural studies - few examples are the studies conducted by de la Sablonnière, Tougas, and Lortie-Lussier (2009), Stoyanova, Doseva, Gergov, and Virginás-Tar (2015), Suls and Mullen (1983-1984). The average age of the population in Europe is growing older (Durand, 1945; Fernández-Ballesteros, 2005; Fernández-Ballesteros, 2006). The growth of elderly populations in Europe is significant (Blotevogel & King, 1996) and it seems important to study mental commitment to the past among this significant growing part of the population.

The longer a person has lived, the stronger the culture may influence the human mentality that is why sentimentality and nostalgia in elderly people should be more influenced by the culture than in youth. This study aims to compare cross-culturally sentimentality and nostalgia among elderly people in Greece and Bulgaria. Gergov and Stoyanova’s (2013) questionnaire measuring sentimentality and nostalgia has been adapted in a Greek sample of elderly people in order to investigate further this issue.

Sentimentality means sensitivity, emotionality (“Sentimental,” 2009; “Sentimental,” 2013). It includes a positive attitude towards the past (Yaybob, 2008). Such definitions make the concept similar to this one of nostalgia.

Johannes Hofer created the term “nostalgia” in 1688 for indicating the pain felt by a person who is not in his/her native land, who yearns for returning home and who fears never to see it again (as cited in “Dying to go home,” 2008; as cited in Scanlan, 2008). Hofer considered as the symptoms of nostalgia sadness, hopelessness, fear, lack of appetite, muscle weakness, sleeping problems, etc. (as cited in “Dying to go home,” 2008; as cited in Scanlan, 2008; as cited in Stefanenko, 1999, pp. 280-282).

Modern theories have developed the notion of nostalgia as related not only to the past memories of the homeland, but to the past in general. The following concepts illustrate this tendency.

- Nostalgia is an attachment to the past (Jobson & Wickham-Jones, 2010), a preference of the experiences, the products (Cattaneo & Guerini, 2012) and the places (Chan, 2011) from the past.
- Nostalgia refers to a longing, a yearning for the past, a fondness for possessions and activities associated with days of yore. Nostalgia involves consumer tastes continuing over time and proneness to patterns of preferences (Holbrook, 1993, p. 245). Nostalgia means that people consider that the products are not made like they used to and that things used to be better in the past (Holbrook, 1993).
- Holbrook and Schindler (1991, p. 330) define nostalgia as a preference, liking, positive attitude, or favourable affect toward people, places, or things that were more common, popular, fashionable, when one was younger.

The past shapes how people determine themselves creating security and self-acceptance (Stamatov, 2010). Elderly people evaluate their abilities applying more frequently temporal comparisons than social comparisons (Suls & Mullen, 1983-1984). There is a growing tendency to re-evaluate one’s life throughout the whole period of old age (Petrov, 1978). This re-evaluation is a purposeful activity aimed at balancing the individual with the environment, besides it increases the value of life experience.

The older people assess more realistically past events (Gradev, 1987) when they consider the influence of the past events on their current life. On the one hand, the presence exercises a kind of pressure on the past. Gradev (1987) conducted a study and 70% of people indicated that significant events for them in the past had lost their original value over time. Gradev (1987) explains this finding by weakening of the initial emotional burden and loss of pathos. Events fade when the present situations require a radical reassessment of the past. In these cases, the memories of the past are deliberately restructured and some separate facts and events that do not conform to the newly living concept are displaced.

Gradev (1987) noted another aspect of the interaction past-present. According to him, there is a mechanism by means of which the present life is a source of enrichment of the past memories. People look for an explanation of their significant life events. Sometimes unconsciously, on a pale memory, they begin to accumulate interpretations and judgments that overexpose the original event beyond its recognition.

Not all events, however, can be a source of sentiment, but only those that are subjectively experienced as significant and carry a positive emotion. According to Georgiev (2003), among the most emotional life events are: graduation, marriage, the childbirth, and success in the professional field. They are the sources of self-esteem and mental stabilization for the individual. The significance of the past life for the elderly people is described by Erickson (1996), who called the last stages of life "integrity versus despair." Depending on the re-evaluation of the previous stages, one gets to enjoy a life of full value or one experiences varying degrees of hopelessness. At this late stage, the results of the previous stages are integrated, and a wise attitude to life follows or the person falls into despair.

Most elders tend to adapt to aging (Daatland, 2003). Good adaptation to old age is expressed as the lack of serious health problems, doing better things based on previous experience, having friends and not feeling lonely and bored, getting on well with one’s family, relatives and friends, planning one’s activities, feeling useful, etc. (Efklides, Kalaitzidou, & Chankin, 2003). Optimal aging includes good health, maintaining high cognitive and physical functioning, emotional balance, and engagement with life (Fernández-Ballesteros, 2005). Successful aging depends on self-efficacy, physical activity, social support and social functioning (Melehin, 2015). Tenacious goal pursuit in old age is important for experiencing positive affects, and flexible goal adjustment is important for escaping negative emotions (Heyl, Wahl, & Mollenkop, 2007). Flexibility is difficult to be achieved by the elderly, because typically they are conservative (Blotevogel & King, 1996). The elderly people are perceived as having poor health and memory impairment, being rigid and inflexible, with low activity (Fernández-Ballesteros, 2006). They need special health care and old-age assistance (Blotevogel & King, 1996). There is a decrease of cognitive functions, in intellectual functioning in old age, often after 65 years old, such as memory diseases, problems with time orientation (Daatland, 2003; Melekhin, 2015), as well as a small decline of extraversion and positive affects, while emotional loneliness and external locus of control increase (Daatland, 2003).

Loneliness is a frequent feeling among elderly people in Greece and Bulgaria (Dykstra, 2009) that suggests some cross-cultural similarities between elderly people in both countries. The cultures in Southern and Eastern Europe are considered more sultry (Gelhaar et al., 2007, p. 150). Southern (especially Latin) crowds claimed to be the most feminine (LeBon, 1896), i.e. sentimental, emotional, etc. Because of this stereotype, sentimentality may be an important part of Greeks and Bulgarians’ life.

Bulgarian and Greek cultures are homogeneous in similar degrees regarding their cultural beliefs and norms of work, family, religion and morality (Uz, 2015). Bulgaria and Greece have similar indexes of individualism/collectivism – 30 and 35 respectfully that means a tendency to collectivism compared with the indexes of USA and Guatemala – 91 (individualistic) and 6 (collectivistic) respectfully (Stavrova, Schlösser, & Fetchenhauer, 2013, pp. 934-935). Greek society has been found to share collectivist values (Efklides, Kalaitzidou, & Chankin, 2003).

The elderly people are among the most vulnerable categories of population who are partially blamed for their situation in the individualist cultures (Hill, Felice, & Ainscough, 2007). As Bulgaria and Greece tend more to collectivism than to individualism, the social attitude towards elderly people in both countries should be more favourable than unfavourable that could slightly decrease the levels of elderly people’s sentimentality and nostalgia to the past.

Greek community has a generalized attitude of support towards the elderly (Caftanzoglou, 1994). Social benefits for elderly population in Greece were bigger than for the non-aged population in the past (Katrougalos, 1996), and other Europeans also demonstrate solidarity towards elderly people seeing them as deserving support and state protection. Informal solidarity towards elderly people is similar in Greece and in Bulgaria (van Oorschot, 2006), but the family solidarity with elderly people is slightly more strongly expressed among Bulgarians than among Greeks (Kankaraš & Moors, 2009).

The trust in social support from other citizens is also similar in Bulgaria and Greece and slightly higher in Greece (Gërxhani & Koster, 2012). The index of state responsibility for social support is similar in Bulgaria and Greece with a slightly higher index of state responsibility for social support in Greece than in Bulgaria (Salmina, 2014). Greek elderly people above 65 years old received more at-home care than their Bulgarian counterparts in the period between 1996 and 2006 (Saraceno, 2010).

The elderly people are claimed to lose from transition process in the society (Marks, Hooghe, Nelson, & Edwards, 2006). The elderly people in Bulgaria as a country of transition of political system could be more sentimental and nostalgic to their past than the elderly people in Greece that is not a country of transition.

Bulgarians perceive income differences in Bulgaria as too large and themselves as not being rewarded enough for their efforts and skills (Suhrcke, 2001). Bulgaria has a high corruption index (Schwartz, 2008). Bulgarian pensioners are the poorest in the European Union. They use the coping strategies of living in multigenerational households; complementing pension income with earnings from small jobs; migrating from urban to rural areas (Asenova & McKinnon, 2007, p. 393). They could experience more sentimentality and nostalgia to the past than Greek elderly people.

Greek pension reform attempted to reduce pensions (Casey, 2012) and this could increase the number of elderly people who need assistance, and respectfully – sentimentality and nostalgia to their past may also increase.

Of course, there is a further differentiation, since there are two types of elderly people – some of them (“young old”) are active and economically independent, the others (“old old”) are poor and submissive, often alone, needing medical care (Blotevogel & King, 1996, p. 138). More and more elderly people work because of the changes in the state policy regarding retirement age (Blotevogel & King, 1996). *Older elderly people could be more sentimental and nostalgic because they are more dependent on their social environment than young olds.*

A study reveals two most typical social representations of the elderly person – Nurturant (courteous, helpful, kind, supportive, friendly, generous, and happy) and Curmudgeon (rude, ill-tempered, mean, greedy, arrogant, selfish, and snobbish) (Liu, Ng, Loong, Gee, & Weatherall, 2003). Theater also stereotypes the elderly people as unkempt, ludicrous, stooped, and functionally disabled (Gamliel, 2012, p. 623).

There are some research findings about the shared stereotypes of elderly people in different countries. The elderly people in the different cultures are commonly perceived as being dependent and needing long-term care (Assous, 2001). They could feel nostalgic for the past independence and autonomy. Loss of physical attractiveness in elderly people in all cultures is a common factor that could contribute to their nostalgia to their past attractiveness.

Women lose more than men their social value by growing old considering their physical appearance, their media portrayal as less successful (Hatch, 2005). That is why *it is expected elderly women to be more sentimental and nostalgic towards their past than elderly men*.

Bulgaria and Greece have similar indices of gender equality (Medalia & Chang, 2011), so *it is expected elderly women to be more sentimental and nostalgic towards their past than elderly men in both countries*.

Satisfaction with life is found to be higher in Greece than in Bulgaria (van de Vliert & Janssen, 2002). That is why it is expected Greek elderly people to be less sentimental and nostalgic towards their past than Bulgarian elderly people.

The expected differences in sentimentality and nostalgia between Bulgarian and Greek elderly people are also supported by some research findings that reveal some cross-cultural difference in several values.

Elderly people in both countries differ in the values like strongly expressed power distance that is more typical for elderly people in Bulgaria than in Greece (Davidkov, 2004). Bulgarians value hierarchy (Schwartz, 2008). Greek culture is more egalitarian than the Bulgarian culture (Schwartz, 2006; Schwartz, 2008).

Bulgarians value tradition (Schwartz, 2006) that is why Bulgarian elderly people could be more sentimental and nostalgic to their past than Greek elderly people.

## Hypotheses of the Study

The main hypothesis of our study was that together with some similarities related to perceived importance of the past for the elderly people in both countries, the elderly people in Bulgaria and Greece would show cross-cultural differences in their sentimentality and nostalgia related to the past such as Bulgarian elderly people would be more sentimental and nostalgic than Greek elderly people.

As stated above, the other variables taken into account are age and gender - it was expected older elderly people to be more sentimental and nostalgic than young olds, and elderly women to be more sentimental and nostalgic towards their past than elderly men.

It was also expected that the questionnaire SNEP would have good psychometric properties (reliability and validity) among Greek elderly people in order to be interpreted the results on it.

## Method

### Sample

The total number of the respondents in Bulgaria and Greece were 283 participants. They participated voluntarily in the study and were purposefully selected to be elderly people above 60 in good mental health (with no previous psychiatric history) and to be able to understand the questions in order to reply. All of the participants in the study were retired.

About 1.3 millions (19.3%) out of the whole Bulgarian population was in the age group 65 years and over (CIA World Factbook, 2014a). About 2.1 millions (20.2%) out of the whole Greek population was in the age group 65 years and over (CIA World Factbook, 2014b). The needed sample size for elderly people in Bulgaria and Greece was 96 in each country (confidence interval 10; confidence level 95%) that was computed by means of an online sample size calculator (Creative Research Systems, 2012).

125 elderly people of Greek origin were studied in 2014 in Greece. The majority of the Greek participants lived with their families in Drama (*N* = 103, of which 50 came from the nearby villages of the Drama prefecture) and some lived in the city of Kavala (*N* = 22). 45 were males (36%) and 60 were females (64%). Their ages ranged from 60 to 89 years old. Their mean age was 72 years old, *SD* = 7 years. The young elders (between 60-75 years old, Lambert-Shute & Fruhauf, 2011, p. 32) were 79 (63.2%) and the oldest old (above 76 years old) were 46 (36.8%). A similar age classification indicates that the elderly people could be differentiated in two age groups - advanced age (55-75 years old) and old age (75-90 years old) (Melekhin, 2015).

158 elderly people of Bulgarian origin (ethnic majority) were studied in 2013 and 2014 in Bulgaria. They lived in Blagoevgrad (*N* = 80), Dobritch (*N* = 20), Kostenets (*N* = 3), Sofia (*N* = 37) and several villages in South-West and North-East Bulgaria (*N* = 18). 61 were males (38.6%) and 97 were females (61.4%). They were from 60 to 92 years old. Their mean age was 73 years old, *SD* = 8 years. The young elders (between 60–75 years old, Lambert-Shute & Fruhauf, 2011, p. 32) were 92 (58.2%) and the oldest old (above 76 years old) were 66 (41.8%). 62 (39.2%) lived with their families. 39 (24.7%) lived alone. 57 (36.1%) lived in institutions for elderly people.

Elderly people living in small or large cities, or villages were not compared, because of their unequal number – a big deal of Bulgarian participants lived in large cities, and because the study that established the psychometric properties of SNEP in the Bulgarian sample of elderly people did not found any statistically significant differences between Bulgarian elders living in different types of places in their nostalgia and sentimentality (Gergov & Stoyanova, 2013).

The participants were not compared also regarding if they lived alone, with their family or in an institution, because all Greek participants lived with their families and such differences have not been found to be statistically significant in the Bulgarian sample of elderly people that was used for establishing the psychometric properties of SNEP when it was created (Gergov & Stoyanova, 2013).

In 2015, another study with 81 elderly people (46 Bulgarians and 35 Greeks) from the same sample was conducted in order to establish test-retest reliability of the questionnaire. They were contacted again and agreed voluntarily to answer the same questionnaire. The period between both tests was about 6 months, so a test-retest reliability coefficient could be computed.

### Instrument

A questionnaire measuring Sentimentality and Nostalgia in Elderly People – SNEP (Gergov & Stoyanova, 2013) was used in Bulgaria in 2013 and 2014. It was translated from English to Greek in 2014 and vice versa. It consisted of 14 items.

In Bulgaria, alpha for the total score was .871; for the scale “Past emotions continue in the present” α = .836; for the scale “Nostalgia of the Past” α = .754; for the scale “Sentimental compensation” α = .678; and for the scale “One’s past perceived by the others” α = .685 (Gergov & Stoyanova, 2013). The total Cronbach’s alpha above .8 is considered to be very good (Price, 2012, p. 117; Wigdor & Green, 1991, p. 118).

The results were statistically processed by means of SPSS 16 applying descriptive statistics for presenting mean tendencies in sentimentality and nostalgia among the elderly in both countries. Factor analysis was performed in the Greek sample for extracting the components of sentimentality and nostalgia and reliability analysis was also performed for adapting the questionnaire in the Greek sample. For group comparisons (gender, age, country), chi square analysis, *t*-test and ANOVA were used.

## Results

### Psychometric Properties of SNEP in Greece

The frequencies of the answers of Greek elderly respondents to every question from SNEP are presented in Table 1.

**Table 1:** Frequency of Agreement With the Items of the Scale Sentimentality and Nostalgia Related to the Past in Elderly People in Greece

Item		Agreement (both strongly agree and agree)		uncertain		disagree	
		*n*	%	*n*	%	*n*	%
1.	The past is very important for me.	109	87.2	14	11.2	2	1.6
2.	The past in a great degree determines my present and my future.	84	67.2	37	29.6	4	3.2
3.	I often think about the past.	99	79.2	17	13.6	9	7.2
4.	When I think about the past, I become full of positive emotions.	63	50.4	37	29.6	25	20.0
5.	The emotions related to the past are a big deal of my present feelings.	106	84.8	18	14.4	1	0.8
6.	I think that the biggest part of the important events in my life have happened in the past.	109	87.2	13	10.4	3	2.4
7.	I am proud of my past.	68	54.4	51	40.8	6	4.8
8.	I would live again my life in the same way.	67	53.6	33	26.4	25	20.0
9.	The past inspires me for the future.	74	59.2	17	13.6	34	27.2
10.	In the past, I have remained unemotional even in situations where most people get very sentimental.	80	64.0	28	22.4	17	13.6
11.	I think that in a certain degree by means of good deeds now I could recompense my past negative acts.	78	62.4	28	22.4	19	15.2
12.	I am more sentimental than most people.	90	72.0	19	15.2	16	12.8
13.	If the people know my past, they estimate me positively	70	56.0	28	22.4	27	21.6
14.	I want the others to know my past	76	60.8	24	19.2	25	20.0

According to the data, the past was very important and related to positive emotions for the majority Greek elderly people (see Table 1).

Factor analysis in the Greek sample was not good enough to be interpreted – KMO = .565, in spite of Bartlett's Test of Sphericity = 304.675, *p* < .001. Because of the poor factor solution, only the total score on the questionnaire measuring sentimentality and nostalgia was interpreted.

Alpha for the total score was .629 indicating that the full version of the scale could be used for measuring Sentimentality and Nostalgia among elderly people in Greece.

In Greece, the test-retest reliability coefficient for a period of six months between the two studies was *r* = .91, *p* < .001. This coefficient is very high and it means not only good psychometric properties of SNEP, but also stability of sentimentality and nostalgia among elderly people in Greece.

The norms on the scale for the Greek sample of elderly people are presented in Table 2. They are in the form of mean and standard deviation, as for the Bulgarian version of the questionnaire SNEP (Gergov & Stoyanova, 2013).

**Table 2:** Norms in the Greek Sample on Total Scale SNEP

	Sentimentality and Nostalgia
*M*	37.53
*SD*	4.71

The same number of the elderly subjects in Greece had low (*N* = 21; 16.8%) and high (*N* = 21; 16.8%) levels of sentimentality and nostalgia. The most Greek elderly people had medium levels of sentimentality and nostalgia (*N* = 83; 66.4%). The elderly Greek respondents with medium levels of sentimentality and nostalgia prevailed over the respondents with high and low levels (χ^2^(2, *N* = 125) = 61.504, *p* < .001).

There were not any significant gender differences on the total score of SNEP in Greece (see Table 3).

**Table 3:** Differences in Sentimentality and Nostalgia Between Genders in Greece

Scale	Age group	*N*	*M*	*SD*	*t*	*df*	*p*
Sentimentality and Nostalgia	Elderly women	80	37.6	4.60	0.188	123	.852
	Elderly men	45	37.4	4.90		

There were not any significant age differences on the total score of SNEP in Greece (see Table 4).

**Table 4:** Differences in Sentimentality and Nostalgia Between Two Age Groups in Greece

Scale	Age group	*N*	*M*	*SD*	*t*	*df*	*p*
Sentimentality and Nostalgia (full form)	Young elders	79	38.10	4.60	1.799	123	.074
	Oldest old	46	36.54	4.79		

Summarizing the findings regarding the psychometric properties of SNEP in Greece, it was found that the questionnaire could be used in its full form without any sub-scales, differing from the Bulgarian version of SNEP that had four sub-scales besides the total score.

### Results From SNEP in Bulgaria

The frequencies of the answers of Bulgarian elderly respondents to every question from SNEP are presented in Table 5. According to the data, the past was also important and related to positive emotions for the majority Bulgarian elderly people (see Table 5).

**Table 5:** Frequency of Agreement With the Items of the Scale Sentimentality and Nostalgia Related to the Past in Elderly People in Bulgaria

Item		Agreement (both strongly agree and agree)		uncertain		disagree	
		*n*	%	*n*	%	*n*	%
1.	The past is very important for me.	117	74.0	29	18.4	12	7.6
2.	The past in a great degree determines my present and my future.	118	74.7	36	22.8	4	2.5
3.	I often think about the past.	134	84.8	13	8.2	11	7.0
4.	When I think about the past, I become full of positive emotions.	99	62.7	47	29.7	12	7.6
5.	The emotions related to the past are a big deal of my present feelings.	95	60.1	40	25.3	23	14.6
6.	I think that the biggest part of the important events in my life have happened in the past.	112	70.9	39	24.7	7	4.4
7.	I am proud of my past.	133	84.2	15	9.5	10	6.3
8.	I would live again my life in the same way.	106	67.0	38	24.1	14	8.9
9.	The past inspires me for the future.	103	65.2	34	21.5	21	13.3
10.	In the past, I have remained unemotional even in situations where most people get very sentimental.	82	52.0	51	32.2	25	15.8
11.	I think that in a certain degree by means of good deeds now I could recompense my past negative acts.	89	56.3	52	32.9	17	10.8
12.	I am more sentimental than most people.	96	60.8	50	31.6	12	7.6
13.	If the people know my past, they estimate me positively	87	55.0	45	28.5	26	16.5
14.	I want the others to know my past	97	61.4	37	23.4	24	15.2

Sentimentality and nostalgia were also stable in Bulgarian elderly people, because the test-retest reliability coefficient for a period of six months between the two studies was *r* = .764, *p* < .001 in Bulgaria.

More elderly subjects in Bulgaria had low (*N* = 36; 22.8%) than high (*N* = 26; 16.5%) levels of sentimentality and nostalgia. The most Bulgarian elderly people had medium levels of sentimentality and nostalgia (*N* = 96; 60.8%). The elderly Bulgarian respondents with medium levels of sentimentality and nostalgia prevailed over the respondents with high and low levels (χ^2^(2, *N* = 158) = 54.43, *p* < .001).

### Cross-Cultural Comparisons Between Sentimentality and Nostalgia in Elderly People in Bulgaria and Greece

Sentimentality and nostalgia in elderly people in both countries were compared only on the total scores of the scale. The answers on each item of the scale were also compared in both countries.

Elderly Bulgarians were more sentimental and nostalgic than Greek elderly people and the effect size was large (see Table 6). The past was more important for Bulgarian elderly people (53.2% strongly agreed) than for the Greek elderly people – 32% strongly agreed (χ^2^(3, *N* = 283) = 37.354, *p* < .001, Phi = .363 – medium effect size). Bulgarian elderly people more often considered that the biggest part of the important events in their life had happened in the past (45.6% strongly agreed) than the Greek elderly people – 35.2% strongly agreed (χ^2^(3, *N* = 283) = 23.786, *p* < .001, Phi = .29 – small effect size). Bulgarian elderly people more often thought about the past (38.6% strongly agreed) than the Greek elderly people – 17.6% strongly agreed (χ^2^(3, *N* = 283) = 15.528, *p* < .05, Phi = .234 – small effect size).

**Table 6:** Differences in Sentimentality and Nostalgia Between the Elderly People in Bulgaria and Greece

Scale	Country	*N*	*M*	*SD*	*t*	*df*	*p*	Cohen’s *d*
Sentimentality and Nostalgia	Bulgaria	158	40.86	7.85	4.423	263**	<.001	1.327
	Greece	125	37.53	4.71				

**Levene’s test was *F* = 48.56, *p* < .001.

Bulgarian elderly people more often considered that the past in a great degree determined their present and future (34.2% strongly agreed) than the Greek elderly people – 5.6% strongly agreed (χ^2^(3, *N* = 283) = 34.04, *p* < .001, Phi = .347 – medium effect size).

Bulgarian elderly people more often declared being proud of their past (44.9% strongly agreed) than the Greek elderly people – 9.6% strongly agreed (χ^2^(3, *N* = 283) = 59.847, *p* < .001, Phi = .46 – medium effect size). Bulgarian elderly people more often would live their lives in the same way (32.3% strongly agreed) than the Greek elderly people – 8.8% strongly agreed (χ^2^(3, *N* = 283) = 25.773, *p* < .001, Phi = .302 – medium effect size). Bulgarian elderly people more often were inspired by the past (30.4% strongly agreed) than the Greek elderly people – 12% strongly agreed (χ^2^(3, *N* = 283) = 22.625, *p* < .001, Phi = .283 – small effect size).

Bulgarian elderly people more often felt positive emotions when they thought about the past (43% strongly agreed) than the Greek elderly people – 10.4% strongly agreed (χ^2^(3, *N* = 283) = 44.315, *p* < .001, Phi = .396 – medium effect size). Bulgarian elderly people more often considered that the emotions related to the past were a significant part of their present emotions (37.3% strongly agreed) than the Greek elderly people – 15.2% strongly agreed (χ^2^(3, *N* = 283) = 67.237, *p* < .001, Phi = .487 – medium effect size).

Bulgarian elderly people more often considered themselves being more sentimental than most people (32.9% strongly agreed) compared to the Greek elderly people – 8.8% strongly agreed (χ^2^(3, *N* = 283) = 47.945, *p* < .001, Phi = .412 – medium effect size). Bulgarian elderly people more often remained unemotional in the past in situations where most people got very sentimental (26.6% strongly agreed) than the Greek elderly people – 6.4% strongly agreed (χ^2^(3, *N* = 283) = 37.14, *p* < .001, Phi = .362 – medium effect size). These findings are not contradictory, because they mean that Bulgarians are now more sentimental, but they considered themselves more unemotional in the past.

Bulgarian elderly people more often thought that in a certain degree by means of good deeds in the present one could recompense his/her past negative acts (19% strongly agreed) than the Greek elderly people – 8% strongly agreed (χ^2^(3, *N* = 283) = 14.295, *p* = .003, Phi = .225 – small effect size). These negative acts should be interpreted mainly in moral terms, because elderly people in all countries are less likely to commit crime than young people and men (in Sun, Sung, & Chu, 2007).

Greek elderly people more often wanted the others to know their past, because there was no reason for shame (20% strongly agreed) than the Bulgarian elderly people – 15.2% strongly agreed (χ^2^(3, *N* = 283) = 31.8, *p* < .001, Phi = .335 – medium effect size). Greek elderly people more often considered that if the others knew their past they would estimate them positively (21.6% strongly agreed) than the Bulgarian elderly people – 16.5% strongly agreed (χ^2^(3, *N* = 283) = 19.599, *p* < .001, Phi = .263 – small effect size). Concerning the opinions about the perception of one’s past by the other people, Bulgarian elderly people were more uncertain and less determined then Greek elderly people.

Oldest olds in Bulgaria were more sentimental and nostalgic (see Table 7) than oldest olds in Greece (p_LSD_ < .001), young elders in Greece (p_LSD_ < .001) and young elders in Bulgaria (p_LSD_ < .001) and the effect size was large.

**Table 7:** Age Differences in Sentimentality and Nostalgia Between the Elderly People in Bulgaria and Greece

Age group and country	*N*	*M*	*SD*	*F*(3, 279)	*p*	Eta squared
Oldest olds in Bulgaria	66	43.86	8.22	15.316	<.001	0.141
Oldest olds in Greece	46	36.54	4.79			
Young elders in Bulgaria	92	38.71	6.85			
Young elders in Greece	79	38.10	4.60			

*Note.* Scale: Sentimentality and nostalgia.

Bulgarian male elders were more sentimental (see Table 8) than Greek male elders (p_LSD_ < .001), Greek female elders (p_LSD_ < .001) and Bulgarian female elders (p_LSD_ = .026), and the effect size was medium. Bulgarian female elders were more sentimental than Greek female elders (p_LSD_ = .02) and Greek male elders (p_LSD_ = .037).

**Table 8:** Gender Differences in Sentimentality and Nostalgia Between the Elderly People in Bulgaria and Greece

Scale	Gender and country	*N*	*M*	*SD*	*F*(3, 279)	*p*	Eta squared
Sentimentality and Nostalgia full form	Men in Bulgaria	61	42.34	7.62	7.573	<.001	0.075
	Men in Greece	45	37.42	4.95			
	Women in Bulgaria	97	39.93	7.90			
	Women in Greece	80	37.59	4.60			

## Discussion

It has been established that the questionnaire SNEP has good psychometric properties in the sample of Greek elderly people. Its reliability in Greece was high enough and it was higher over time – test-retest reliability, than across items – internal consistency. In the sample of Greek elderly people, Cronbach’s alpha of .629 means marginal reliability (between .6 and .7, according to Wigdor & Green, 1991, p. 118) and test – retest reliability of .91 means very good reliability (above .8, according to Price, 2012, p. 117) or excellent reliability (above .9, according to Wigdor & Green, 1991, p. 118). The test – retest reliability of .764 in Bulgarian elderly people is acceptable (between .7 and .8, according to Wigdor & Green, 1991, p. 118). These results mean a tendency of stability in expression of sentimentality and nostalgia in elderly people for a six-month period, especially in Greek elders.

Concerning construct validity of SNEP, its factor structure has not been confirmed in the Greek sample, because of poor adequacy of the factor model revealed by KMO index, and only the total score on SNEP has been used for intergroup comparisons, but the theoretical model that expected some cross-cultural, age and gender differences was confirmed. The theoretical model expecting higher sentimentality and nostalgia among Bulgarian elders than among Greek elders, as well as among older elderly people (Bulgarians as indicated the results) compared with young elders (Greek and Bulgarians, according to the results) was confirmed. It has been found that Bulgarian female elders were more sentimental than Greek male elders, as the theoretical model for construct validity expected, but Bulgarian male elders were more sentimental than Bulgarian and Greek female elders.

Traditionally the stereotypes of women describe them as more emotional, irrational, and incompetent than men (Secker, 1999). Men often are associated with qualities such as activity, instrumentality, and determination (Dragneva, 1990; Kotseva & Todorova, 1994). Men seem to have suffered more in retirement, because they lost their utility in their job that was a way of being valuable and significant, while the women seem not to be able to escape the obligations of housewife, and still feel responsible for harmony and order at home (Alexandrova, 2006, p. 273). Loss of different professional and social activities by men could make them more nostalgic to the past when they felt themselves more useful and valuable, so their sentimentality increased as they got older, poorer and socially excluded – like Bulgarian oldest old men. Socialization factors could make women become more sentimental like Bulgarian elderly women compared to elderly people in Greece. One cross-cultural study has already revealed more emotionality (higher anxiety and sad emotions) in Bulgarian women than in Greek women (Giannouli & Ivanova, 2016).

The main hypothesis of the study was confirmed. In both countries, the elderly people with medium level of sentimentality and nostalgia prevailed and the past was important for them. On the other side, the elderly people in Bulgaria and Greece differed significantly in their sentimentality and nostalgia related to the past. The differences were expressed mainly in the direction of more increased values of the studied phenomena in the Bulgarian sample. These differences could be due to many factors. One of them is the different social-economic status of Bulgarian and Greek pensioners. The mean pension in Bulgaria is about 159 Euro (*National Social Security Institute*, 2014), while in Greece it is about 942 Euro (Vasileva, 2014). Besides, 35% of Greek pensioners receive a second pension, and 12% of them receive even a third pension (Blitz, 2014; GR Reporter, 2014). Retail expenditures per capita are higher in Greece than in Bulgaria, related to economic freedom, access and availability (Kshetri & Bebenroth, 2012). These differences in the standard reflect on the attitudes towards the past. In Bulgarian pensioners, they may have played an important role on the high levels of nostalgia and sentimentality.

The sudden change of income after retirement makes difficult for a lot of people to survive, and this in turn affects the subjective assessment of the past, giving it an outstanding value. The low income makes impossible for older people to maintain their previous social activity and it contributes to their social exclusion. The new standard imposes some limits of any kind and the past becomes more and more important.

Another possible explanation of the resulting differences is the transition of Bulgarian society between two economic systems during the last twenty-five years. This transition lasted extremely long and it was painful for a significant part of the population. It was accompanied by disturbances of all levels. Uncertainty has appeared in the society. The value system has changed. The primordial attitudes and perceptions devaluated. The aging personality has difficulties to adjust oneself to new realities. A peculiar fixation on the past is the result from this maladaptation, on the time when the personality was formed and realized. The longer a person has lived in a socio-economic system, the more affectionate and sentimental s/he is to this system. The past is more and more often compared to the present and there are some possible expectations of reversibility of the parameters describing the old times.

A cross-cultural study among the elderly people revealed that Greek elders received more social support, especially family support compared to the elders in Portugal, Italy, Germany, Spain and Sweden (Melchiorre et al., 2013). A part of the Bulgarian participants in this study lived in institutions for elderly people, while no Greek respondent lived in such an institution. This difference between the samples in both countries could also explain why the Bulgarian elders were more sentimental and nostalgic towards their past than the Greek elders, who continued feeling the support by their families.

Higher sentimentality and nostalgia among Bulgarian elderly people compared with Greek elders could be related to higher depression because of not feeling family support in institutions. As some authors state, lack of social integration of individuals because of divorce and absence of children raises the suicide rate, whereas marriage lowers it (Neumayer, 2003). Ex-communist nations have high suicide rate related to lower self-expression (Lenzi, Colucci, & Minas, 2012). The different social and economic conditions of the elders in both countries were one of the limitations of this study.

Future studies of sentimentality and nostalgia in elderly people should focus on the cognitive factors which may influence the way that elders think about the past and at the same time they should clarify the stereotypes related to ageism. This future research will prove if there is a cognitive explanation for the nostalgia and sentimentality in elders or if the possible differences in the stereotypes towards elders in both countries are solely responsible for the way that the elders see and feel about their current and previous personal history.

The established tendency for stability of sentimentality and nostalgia in elderly people for a six-month period could be studied further in longitudinal research.

The psychometric properties of the questionnaire SNEP may be studied further in different samples and populations that would permit other cross-cultural comparisons.
